# Distinct Inflammatory Immune Signature in Acute Mpox Associated With Proctitis

**DOI:** 10.1093/ofid/ofag460

**Published:** 2026-07-23

**Authors:** Joanne Byrne, Gurvin Saini, Alejandro Garcia-Leon, Dana Alalwan, Alan Landay, Liem Binh Luong Nguyen, Stefano Savinelli, Cathal O’Broin, Mary Horgan, Christine Kelly, Aoife Cotter, Corinna Sadlier, Eoghan de Barra, Jane A O’Halloran, Virginie Gautier, Patrick W G Mallon, Eoin R Feeney

**Affiliations:** Centre for Experimental Pathogen Host Research, University College Dublin, Dublin 4, Ireland; Department of Infectious Diseases, St Vincent's University Hospital, Dublin 4, Ireland; Centre for Experimental Pathogen Host Research, University College Dublin, Dublin 4, Ireland; Centre for Experimental Pathogen Host Research, University College Dublin, Dublin 4, Ireland; Centre for Experimental Pathogen Host Research, University College Dublin, Dublin 4, Ireland; Department of Medicine, University of Texas Medical Branch, Galveston, Texas, USA; Institut Pasteur, Université Paris Cité, Paris, France; CIC Cochin Pasteur, Assistance Publique-Hôpitaux de Paris, Paris, France; Centre for Experimental Pathogen Host Research, University College Dublin, Dublin 4, Ireland; Department of Infectious Diseases, St Vincent's University Hospital, Dublin 4, Ireland; Centre for Experimental Pathogen Host Research, University College Dublin, Dublin 4, Ireland; Department of Infectious Diseases, St Vincent's University Hospital, Dublin 4, Ireland; School of Medicine, University College Dublin, Dublin 4, Ireland; Department of Infectious Diseases, Mater Misericordiae University Hospital, Dublin 7, Ireland; Centre for Experimental Pathogen Host Research, University College Dublin, Dublin 4, Ireland; Department of Infectious Diseases, Mater Misericordiae University Hospital, Dublin 7, Ireland; Centre for Experimental Pathogen Host Research, University College Dublin, Dublin 4, Ireland; Department of Infectious Diseases, Mater Misericordiae University Hospital, Dublin 7, Ireland; Department of Infectious Diseases, Cork University Hospital, Cork, Ireland; Department of Infectious Diseases, Beaumont Hospital, Dublin 9, Ireland; Department of International Health and Tropical Medicine, Royal College of Surgeons in Ireland, Dublin 2, Ireland; Centre for Experimental Pathogen Host Research, University College Dublin, Dublin 4, Ireland; Department of Infectious Diseases, St Vincent's University Hospital, Dublin 4, Ireland; Centre for Experimental Pathogen Host Research, University College Dublin, Dublin 4, Ireland; Centre for Experimental Pathogen Host Research, University College Dublin, Dublin 4, Ireland; Department of Infectious Diseases, St Vincent's University Hospital, Dublin 4, Ireland; Centre for Experimental Pathogen Host Research, University College Dublin, Dublin 4, Ireland; Department of Infectious Diseases, St Vincent's University Hospital, Dublin 4, Ireland

**Keywords:** inflammation, monkeypox virus, mpox, proctitis

## Abstract

**Background:**

The immunopathogenesis of clade IIb monkeypox virus (MPXV) infection and its relationship with clinical severity remain poorly defined. We characterized cytokine responses in acute and convalescent mpox to delineate inflammatory profiles.

**Methods:**

In a multicenter cohort, we quantified 45 plasma protein biomarkers in 3 adult groups: acute mpox (polymerase chain reaction–confirmed clade IIb MPXV infection sampled <14 days from symptom onset); convalescent mpox (>30 days postinfection); and uninfected controls matched for age, sex, race, and HIV status. Biomarkers spanned innate and adaptive immune activation, systemic inflammation, and tissue repair. Principal component analysis and unsupervised hierarchical clustering defined immune profiles. Associations with clinical features were explored via logistic regression.

**Results:**

Sixteen participants with acute mpox (median [IQR], 6 days [4–8] from symptom onset; 13% vaccinated) were compared with 16 controls. Three immune clusters were identified. Cluster 1 comprised only unvaccinated acute mpox and exhibited a broad inflammatory signature (IFN-γ, CXCL9/10/11, CCL 7/8, IL-6/10, OSM). Cluster 2 displayed elevated TNFSF12 and FLT3LG, suggestive of tissue remodeling. Cluster 3 demonstrated lower cytokine expression. Mucosal involvement was associated with the inflammatory cluster 1 (odds ratio, 7.85; 95% CI, 1.02–102.40; *P* = .048). Among 25 participants who were convalescent (median [IQR], 405 days [225–450] postinfection; 12% vaccinated), clustering did not distinguish cases from controls. However, targeted comparisons demonstrated persistent low-grade immune activation (IL-6, OSM) and tissue-remodeling signatures (TGF-α, VEGF-A).

**Conclusions:**

Acute clade IIb mpox displays distinct inflammatory profiles, with systemic inflammation concentrated among those with mucosal disease. Convalescence shows partial resolution but persistent tissue repair activity. These pathways may support risk stratification and targeted therapeutic interventions.

Monkeypox virus (MPXV) is a zoonotic orthopoxvirus (OPXV), with human cases reported in Central and West Africa since 1970 [1]. Two genetically distinct clades are recognized. Historically, clade I has been associated with higher reported case fatality rates than clade II, although disease severity is increasingly recognized as context dependent and influenced by host and epidemiologic factors [2, 3]. In 2022, a major epidemiologic shift occurred with the emergence of clade IIb, characterized by sustained human-to-human transmission, predominantly through close physical and intimate contact, particularly within sexual networks [4]. This outbreak exceeded 100 000 reported cases globally and prompted the World Health Organization to declare a public health emergency of international concern [5]. A second declaration followed in 2024, driven by escalating clade I transmission in Central Africa, where different epidemiologic risk factors and, in some settings, higher mortality were observed [6].

The clinical presentation of the 2022 clade IIb outbreak differed substantially from historical descriptions. Lesions were predominantly anogential, with frequent mucosal involvement, particularly proctitis. While mucosal involvement has been reported in prior outbreaks, including the 2017 reemergence in Nigeria, the predominance of anogenital lesions and proctitis appears distinctive to the 2022 outbreak [4, 7–9]. Genomic analyses revealed an enrichment of APOBEC3-associated mutations in lineage B viruses, a sublineage of clade IIb mpox viruses that emerged during the 2022 outbreak, consistent with prolonged replication in human hosts, although implications for viral fitness and host-immune interactions remain unclear [10]. Despite the unprecedented scale of transmission, overall disease severity was low, with pooled estimates indicating a case hospitalization rate of 5.8% and case fatality rate <0.1%, occurring predominantly in young adults, in contrast to historical clade I outbreaks where cases were more common in pediatric populations [5, 11]. However, people with HIV (PWH) were disproportionately affected, and advanced HIV infection was strongly linked to severe outcomes [7, 12].

Differences in immunopathogenesis and host-pathogen interactions are believed to underpin the heterogeneity in presentation and outcome to OPXV disease. In smallpox, severe and fatal disease was associated with dysregulated host responses resembling a cytokine storm based on retrospective pathologic analyses and nonhuman primate models of variola virus infection [13, 14]. Similarly, in clade I mpox, cytokine profiling has implicated dysregulated inflammatory responses in severe disease, including elevations in IL-2R, IL-10, and GM-CSF [15]. In contrast, the inflammatory response to clade IIb mpox remains poorly defined, with existing studies failing to identify consistent inflammatory signatures or establish correlations with clinical outcomes [16–18].

Effective therapeutic options for mpox remain limited. Although tecovirimat, an antiviral targeting the VP37 protein is currently available, recent randomized trials have demonstrated no clinical benefit in clade I or II disease [19–21]. These findings highlight the urgent need to explore alternative treatment strategies. Increasing evidence supports the role of host-directed interventions rather than antivirals, with modulation of inflammatory pathways proven to reduce morbidity and mortality in viral infections such as COVID-19 [22].

Extending beyond acute mpox infection, even less is known about the immune landscape following recovery from mpox. While inflammation is expected to resolve with recovery, reports of persistent fatigue and ongoing anal pain raise the possibility of sustained immune activation [23–25]. To address these gaps, we characterized cytokine, chemokine, and growth factor responses in acute and convalescent clade IIb mpox, aiming to define inflammatory profiles and their associations with clinical disease.

## METHODS

### Patient Consent Statement

The All-Ireland Infectious Diseases Cohort Study is a prospective multicenter observational study, which was approved by the National Research Ethics Committee in Ireland (reference 20-NREC-COV-056). Adult participants provided informed written consent for the collection of clinical data and blood samples for biobanking.

### Study Design and Participants

For the acute analysis, participants with polymerase chain reaction–confirmed clade IIb MPXV infection were included if sampled within 14 days of symptom onset. Samples were collected prior to initiation of corticosteroids, nonsteroidal anti-inflammatory drugs, or other analgesics; participants had taken paracetamol only prior to sampling. Controls were prevaccination gay, bisexual, and other men who have sex with men without prior mpox, matched 1:1 to cases by age, sex, race, and HIV status. For the convalescent analysis, samples collected >30 days after symptom onset were included and compared with the same control group. Participants were sampled between June 2022 and February 2024.

Data were collected at the time of sample collection on HIV status, CD4^+^ T-cell count, and vaccination history. Vaccinated participants had received 2 doses of the MVA-BN vaccination (JYNNEOS) per national immunization guidelines, and none had received childhood smallpox vaccine [26]. Clinical severity was assessed by the Mpox Severity Score System (Mpox-SSS) [27].

### Cytokine Analysis

We quantified 45 protein biomarkers from plasma using the Olink Target 48 Cytokine inflammation panel (Olink Proteomics). Biomarkers encompassed markers of innate immune activation (IL-6, CXCL8, IL-15, IL-17A, IL-17C, IL-17F, IL-18, CCL2, CCL3, CCL4, CCL7, CCL8, CXCL9, CXCL10, CXCL11, TNF, CSF1, CSF2, CSF3), Th1 activation (IL-1β, IL-2, IFN-γ), Th2 activation (IL-4, IL-13, IL-33, TSLP, CCL11, CCL13), endothelial activation (VEGF-A, CXCL12), tissue repair (OSM, EGF, HGF, TGF-α, FLT3LG), immune regulation (IL-7, IL-10, IL-27, LTA, CCL19), matrix remodeling and inflammation (MMP1, MMP12), and metabolic oxidative stress and modulation (OLR1, TNFSF10, TNFSF12). Protein concentrations were reported in picograms per milliliter (pg/mL).

### Statistical Analysis

Categorical variables were summarized as frequencies and compared with the χ^2^ test. Continuous variables were summarized by median and IQR and compared with the Mann-Whitney *U* or Kruskal-Wallis test with Dunn post hoc comparisons. *P* values were adjusted for multiple comparisons via Benjamini-Hochberg false discovery rate adjustment.

Inflammatory profiles were identified by hierarchical clustering on principal components (PCs). Biomarkers were log transformed, and PC analysis was used for dimensionality reduction. The number of PCs retained were automatically selected by the hierarchical clustering on PCs algorithm to reflect the optimal number of PCs based on internal variance structure. Next, we performed cluster analysis to identify groups of similar observations. Unsupervised hierarchical clustering was performed on the retained PCs by using Ward minimum variance linkage and squared euclidean distance. The number of clusters was chosen by calculating the within-cluster sum of squares (inertia) for each solution and cutting the dendrogram at the point with the greatest loss of inertia. *K*-means consolidation was performed to minimize within-cluster variance.

To examine associations between clinical features and inflammatory cluster membership, we used Firth penalized logistic regression to account for small sample size and potential separation. Models were limited to single covariate adjustment given the sample size. Mucosal involvement was coded as a binary variable (present vs absent). In the acute dataset, we assessed whether clinical characteristics were associated with cluster assignment. In the convalescent dataset, we evaluated whether belonging to the convalescent group (vs control) or having prior mucosal involvement was associated with cluster membership. All statistical analyses were performed in R software (version 4.4.1).

## RESULTS

### Study Population

A total of 16 participants with acute mpox (100% male sex; median [IQR] age, 33 years [30–39]; sampled at a median 6 days [4–8] from symptom onset) were compared with 16 matched controls (Table 1). Within the acute mpox group, 6% (1/16) were PWH (HIV-1), and 13% (2/16) represented breakthrough infections following prior 2-dose MVA-BN vaccination. The participant with HIV was virally suppressed at the time of sampling, with a CD4^+^ T-cell count of 677 cells/µL (37%). Disease severity scores ranged from 4 to 13, with a median Mpox-SSS score of 8.5 (5–10; Table 1). Concurrent sexually transmitted infection screening was performed at presentation in 14 of 16 participants for *Chlamydia trachomatis* and *Neisseria gonorrhoeae* and in all participants for herpes simplex virus and syphilis, which identified no active coinfections. HIV status was ascertained in all participants at enrollment. Three participants reported sexually transmitted infections within the preceding 3 months, all of which had been treated >30 days prior to mpox presentation with negative testing at enrollment. No participants had evidence of systemic bacterial superinfection at sampling; local secondary skin infection was noted in a minority without systemic features.

**Table 1.** Demographics of Study Population

	Median (IQR) or No. (%)		
	Acute Mpox (n = 16)	Matched Controls (n = 16)	Convalescent Mpox (n = 25)
Age, y	33 (30–39)	31 (29–39)	33 (30–40)
Male sex at birth	16 (100)	16 (100)	25 (100)
Race: Caucasian	16 (100)	16 (100)	25 (100)
People with HIV	1 (6)	1(6)	4 (16)
Mpox parameters			
Vaccinated	2 (13)	…	3 (12)
Days from symptom onset	6 (4–8)	…	405 (225–450)
Overall severity score ^a^	8.5 (5–10)	…	8.5 (7.75–9.25)

^a^Mpox Severity Scoring System: sum of scores ranges from 1 through 23 [27].

The convalescent analysis included 25 participants (100% male sex; median [IQR] age, 33 years [30–40]; sampled at a median 405 days [225–450] from symptom onset), which was compared with the same control group (Table 1). Of the 16 individuals included in the acute analysis, 14 were also included in the convalescent cohort. In this analysis, 16% (4/25) were PWH and 12% (3/25) had postvaccination breakthrough infections. The median Mpox-SSS score was 8.5 (7.75–9.25). No hospitalizations or deaths occurred in either group.

### Distinct Inflammatory Signature in Acute Mpox Infection

**Figure 1.**
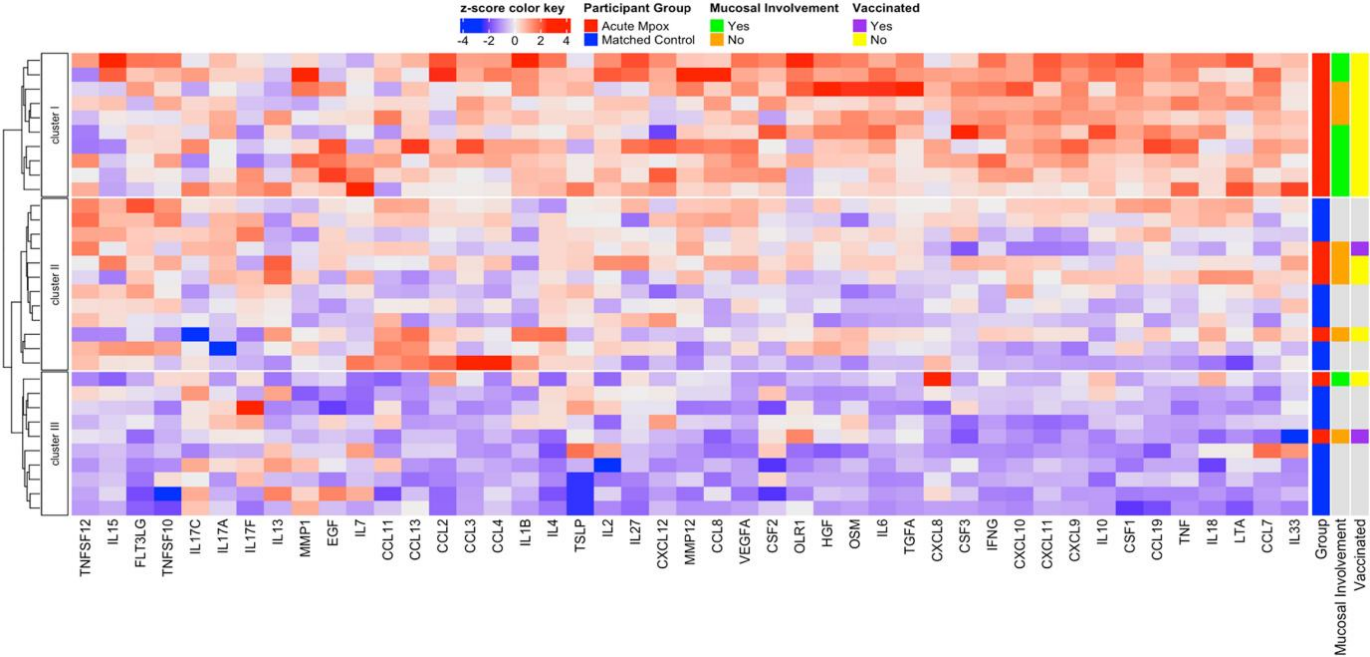
Distinct inflammatory clusters in acute mpox. The heat map displays unsupervised hierarchical clustering of all participants based on *z* score–transformed levels of 45 plasma protein biomarkers. Rows represent individual participants, and columns represent inflammatory biomarkers. Red indicates higher and blue indicates lower relative biomarker expression. Right-hand annotation denotes participant group.

For the acute mpox analysis, PC analysis on the 45 biomarkers retained 6 PCs, accounting for 71.10% of the total variance (scree plot is shown in Supplementary Figure 1). Hierarchical clustering identified 3 distinct clusters: cluster 1, 10 participants (31%; 100% acute mpox); cluster 2, 12 participants (38%; 33% acute mpox); and cluster 3, 10 participants (31%; 10% acute mpox; Figure 1). Clinical characteristics of these clusters are shown in Table 2.

**Table 2.** Participant Characteristics Across Inflammatory Clusters in Acute Mpox and Matched Controls

	Median (IQR) or No. (%)			
Cluster Demographics	Cluster 1 (n = 10)	Cluster 2 (n = 12)	Cluster 3 (n = 10)	*P* Value
Age, y	33 (28.5–42.5)	32.5 (29.5–38)	30.5 (29.2–38.8)	.81*
Male sex at birth	10 (100)	12 (100)	10 (100)	…
Race: Caucasian	10 (100)	12 (100)	10 (100)	…
People with HIV	1 (10)	0 (0)	1 (10)	.53**
Group				
Acute mpox	10 (100)	4 (33)	2 (20)	
Matched control	0 (0)	8 (67)	8 (80)	.0006**
**Acute mpox**				
Vaccinated	0 (0)	1 (25)	1 (50)	.10**
Days from symptom onset	5 (3–6.75)	8 (5.5–10.8)	11 (10–12)	.06*
Overall severity score ^a^	8.5 (5.25–9.75)	7 (4.25–9.25)	7 (5–9)	.77*
Active lesion burden, No.	1.5	1.5	1.5	
Lesion burden, extent of body involvement	1.5	2	1.5	
Confluent lesion or lesions with diameter >2 cm	2	1	1	
Treatment for bacterial superinfection	0	0	0	
Mucosal areas affected	1.5	0	1	
Level of care	1	1	1	
Pain, analgesia requirement	1	0.5	1	

^a^Mpox Severity Scoring System: sum of scores ranges from 1 through 23 [27]. Subcategory data are presented as median.

^*^ *P* value as derived from Kruskal-Wallis test.

^**^ *P* value as derived from χ^2^ test.

Cluster 1 comprised only unvaccinated participants, all with acute mpox, sampled at a median 5 days (IQR, 3–7) from symptom onset. This cluster showed a broad inflammatory signature with elevated markers of innate immune function (IL-6, CXCL9, CXCL10, CXCL11, CCL7, CCL8, TNF, CSF1), Th1 responses (IFN-γ, IL-1B), immune regulation (IL-10, CCL19), endothelial function (VEGF-A), and tissue repair (OSM, TGF-α).

In contrast, cluster 2 included only 4 participants (33%) with acute mpox (1 vaccinated) sampled at a median 8 days (IQR, 6–11) from symptom onset and 8 matched controls. This cluster was characterized by upregulation of markers of tissue repair and dendritic cell function, notably TNFSF12 (TWEAK) and FLT3LG, consistent with a transition toward immune modulation and tissue remodeling.

Last, cluster 3 comprised only 2 participants (20%) with acute mpox (1 vaccinated) sampled at a median 11 days (IQR, 10–12) from symptom onset and 8 negative controls. This cluster had significantly lower levels of biomarkers of innate immune function (IL-6, IL-18, TNF, CXCL10, CCL3, CCL8), Th1 responses (IFN-γ), Th2 responses (IL-4, CCL13), immune regulation (CCL19, TNFSF10), endothelial function (VEGF-A), and tissue repair (HGF, FLT3LG). The pattern reflects a relatively lower overall inflammatory state, with dampened responses across proinflammatory and regulatory pathways as compared with the other clusters.

Individual biomarker analysis confirmed that key markers defining cluster 1 were also elevated in acute mpox as compared with controls, supporting this profile as a broader signature of host responses to acute mpox (Supplementary Table 1, Supplementary Figure 2).

### Cytokine Modulation Associated With Clinical Manifestations of Mpox

Despite the different inflammatory profiles, demographics were similar across the 3 clusters (Table 2). Time from symptom onset was shorter in cluster 1 (median, 5 days) than in clusters 2 (8 days) and 3 (11 days), although this was not statistically significant (*P* = .06). No individuals in the inflammatory cluster 1 had received the MVA-BN vaccination, as compared with 1 participant in cluster 2 (25%) and 1 in cluster 3 (50%; *P* = .10). Although mpox disease severity scores were comparable across clusters, the prevalence of mucosal involvement, all presenting as proctitis, varied across clusters with 7 participants in cluster 1 (70%) affected, no participants in cluster 2 (0%), and 1 participant in cluster 3 (50%; *P* = .06).

Mucosal involvement was significantly associated with the inflammatory cluster 1 (odds ratio [OR], 7.85; 95% CI, 1.02–102.40; *P* = .048). The magnitude of this association was not notably attenuated after adjustment for time since symptom onset, though no longer statistically significant (adjusted OR, 9.49; 95% CI, .75–1931.81; *P* = .09). This effect persisted after adjustment for age, with a similar increase in odds of cluster 1 membership, although the association again was not statistically significant (adjusted OR, 8.26; 95% CI, .90–195.83; *P* = .06).

### Inflammatory Profiles in Convalescent Cases

**Figure 2.**
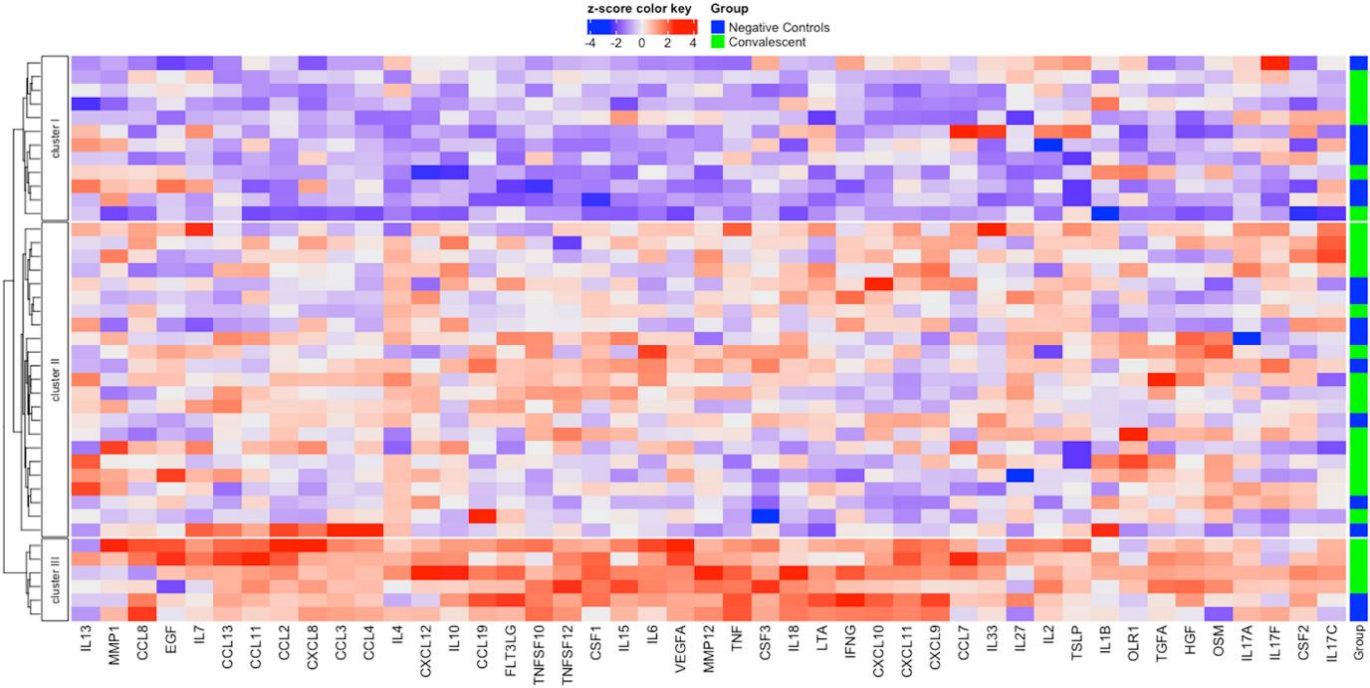
Unsupervised hierarchical clustering of plasma cytokine profiles in convalescent mpox and negative controls. The heat map displays unsupervised hierarchical clustering of all participants based on *z* score–transformed levels of 45 plasma protein biomarkers. Rows represent individual participants, and columns represent inflammatory biomarkers. Red indicates higher and blue indicates lower relative biomarker expression. Right-hand annotation denotes participant group.

In analysis of the 25 participants who were convalescent as compared with 16 controls, PC analysis retained 6 PCs, accounting for 66.85% of the total variance (scree plot is shown in Supplementary Figure 3). Hierarchical clustering again identified 3 distinct clusters (Figure 2, Supplementary Table 2): cluster 1, 12 participants (50% convalescent, sampled at a median 396 days [IQR, 376–448] after symptom onset); cluster 2, 23 participants (65% convalescent, sampled at a median 410 days [227–441] after symptom onset); and cluster 3, 6 participants (67% convalescent, sampled at a median 290 days [119–470] after symptom onset). Demographics, HIV status, mpox severity scores, time since infection, and vaccination status did not differ significantly across clusters. Clinical characteristics of these clusters are shown in Supplementary Table 2.

Cluster 1 included 6 convalescent cases and 6 controls and displayed reduced biomarkers of innate immune function (IL-6, IL-18, TNF, CXCL10, CCL3, CCL8, CSF1), Th1 (IFN-γ), Th2 (IL4, CCL13), immune regulation (CCL19, TNFSF10), endothelial function (VEGF-A), and tissue repair (HGF, FLT3LG). This profile suggests that a low inflammatory state is present in convalescent cases and controls in this cluster. Cluster 2 included 15 convalescent cases and 8 negative controls and showed selective upregulation in markers of tissue repair (FLT3LG) and immune regulation (TNFSF12), suggesting a state of partial immune activation with features of tissue remodeling and regulation.

Cluster 3 represented a highly inflamed profile, characterized by increases in markers of innate immune function (IL-6, CXCL9, CXCL10, CXCL11, CCL7, CCL8, TNF, CSF1), Th1 (IFN-γ, IL1β), immune regulation (IL-10, CCL19), endothelial function (VEGF-A), and tissue repair (OSM, TGF-α). Participants in this cluster were sampled at a median 290 days (IQR, 119–470) from symptom onset, and all were unvaccinated.

Belonging to the convalescent group was not associated with an increased likelihood of being in the inflamed cluster (OR, 0.82; 95% CI, .13–4.28; *P* = .82). In contrast to acute mpox, in convalescent cases, prior mucosal involvement was not associated with membership of the inflamed cluster 3 as compared with cluster 1 or 2 (OR, 1.47; 95% CI, .20–17.10; *P* = .71).

### Persistent Cytokine Signatures in Convalescent Mpox

**Figure 3.**
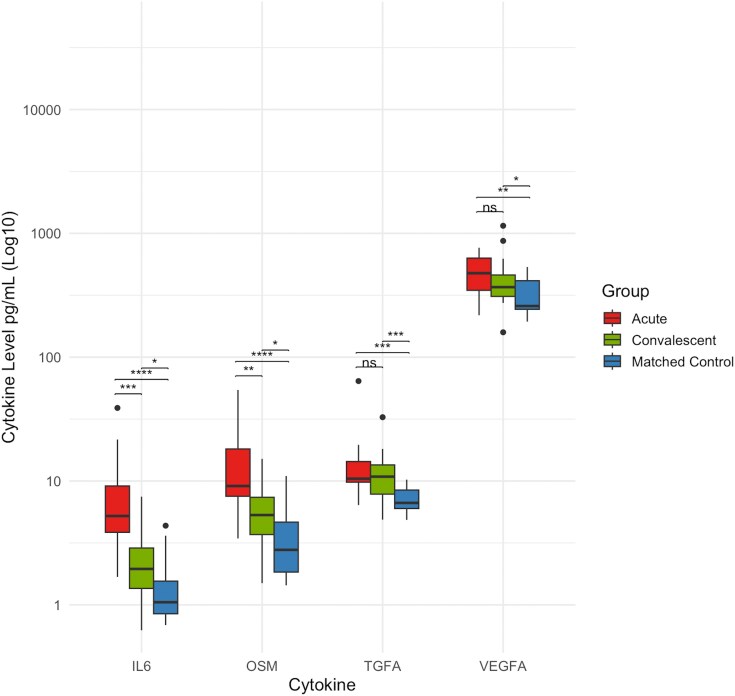
Differential cytokine expression in acute and convalescent mpox as compared with negative controls. Box plots show log_10_-transformed plasma levels of IL-6, OSM, TGF-α, and VEGF-A across acute, convalescent, and matched control groups. Data are presented as median (line), IQR (box), 95% CI (error bars), and outliers (circles). Horizontal bars indicate statistical comparisons (Mann-Whitney *U* test). *P < .05. **P < .01. ***P < .001. ****P < .0001. ns, not significant.

As clustering analyses did not reveal a distinct convalescent signature, we compared cytokine levels across the acute, convalescent, and control groups. Differential expression was observed for 4 cytokines (Supplementary Table 3). IL-6 and OSM remained elevated in convalescence as compared with negative controls, with median concentrations of 1.95 pg/mL (IQR, 1.36–2.88) vs 1.05 (0.85–1.57) for IL-6 and 5.31 pg/mL (3.70–7.39) vs 2.78 (1.84–4.66) for OSM (*P* = .04 and *P* = .03, respectively). However, both cytokines were significantly lower than in acute mpox, where IL-6 measured a median concentration of 5.24 pg/mL (3.85–9.12) and 9.12 (7.55–18.29) for OSM (*P* = .001 and *P* = .01; Figure 3). This pattern suggests partial resolution of much of the acute inflammatory response with persistence of low-grade immune activation. In contrast, TGF-α and VEGF-A were elevated in acute and convalescent samples as compared with controls (all *P* < .05) and showed no difference between phases, consistent with a sustained tissue remodeling signature beyond acute infection.

Given the association between proctitis and the inflammatory profile in the analysis of acute cases, we examined whether prior proctitis influenced cytokine levels in convalescence. Only IL-13 differed between groups. Participants with prior proctitis had significantly lower IL-13 concentrations (n = 16; median, 0.28 pg/mL [IQR, 0.15–0.54]) than those without prior proctitis (n = 9; 8.92 pg/mL [1.73–16.10]; *P* = .02).

## DISCUSSION

This study is the first to define systemic inflammatory profiles in clade IIb mpox, identifying a distinct inflammatory profile in acute infection associated with mucosal involvement (proctitis) and evidence of increases in markers of immune activation and tissue remodeling beyond 1 year postinfection. The acute inflammatory profile was characterized by IFN-γ–driven chemokine activation with broad innate immune and tissue repair signaling. That this was associated with mucosal disease, particularly proctitis, and occurred only in unvaccinated participants suggests a context-specific host-pathogen interaction at the mucosal interface that may contribute to observed clinical heterogeneity in clade IIb mpox (Supplementary Figure 4). These findings provide new insight into the immunopathogenesis of clade IIb mpox. Importantly, they reflect predominantly mild nonhospitalized clade IIb infection in an immunocompetent adult cohort and should be interpreted within this clinical context. While clade I disease in Central Africa has been linked to responses skewed toward Th2 and IL-10 responses, with elevated GM-CSF and sIL-2R [15], our data suggest a more IFN-γ–dominant profile in clade IIb. Several virologic and epidemiologic factors may account for this divergence. First, genomic analyses of B.1 lineage viruses within clade IIb have demonstrated APOBEC3-associated mutational signatures, consistent with sustained human transmission and ongoing adaptation to human hosts [10]. These mutational pressures may influence viral immunomodulatory gene function, although direct functional consequences have not been fully established. In addition, host populations and exposure routes differ markedly across settings: the only clade I cohort with inflammatory responses characterized to date comprised predominantly pediatric cases in Central Africa, with many infections linked to zoonotic exposure [15], in contrast to our adult cohort in which clade IIb infection occurred exclusively through mucosal exposure within sexual networks. These factors may jointly modulate host-pathogen interactions. Nevertheless, the distinct inflammatory landscapes observed across mpox clades underscore the importance of clade-specific immune characterization, which may help explain the differences in clinical manifestations.

Inflammation in acute clade IIb mpox infection was strongly associated with mucosal involvement in our analysis. Proctitis has been one of the most distinctive and burdensome manifestations of the clade IIb outbreak, frequently requiring hospitalization for symptom control despite otherwise mild systemic disease [24]. Our findings suggest that mucosal disease may act as a driver of systemic immune activation and contribute to morbidity. This raises the possibility that targeted modulation of anti-inflammatory pathways could reduce symptom burden in affected individuals, even when systemic disease is otherwise mild.

Our data suggest a potential immunomodulatory role of vaccination. The inflammatory profile identified in acute infection occurred exclusively in unvaccinated individuals, while vaccinated individuals were distributed across lower inflammatory clusters. Although underpowered to support causal inference, this pattern is consistent with clinical observations of attenuated disease following vaccination [28, 29]. Larger studies will be required to define the impact of vaccination on host inflammatory profiles in mpox. It also raises important questions about the longevity of vaccine-mediated immune modulation and whether vaccination could influence host inflammatory responses long after administration despite observed waning antibody titers [30].

During convalescence, participants did not segregate into distinct inflammatory clusters as compared with controls, but direct comparisons of individual biomarkers identified modest but persistent alterations in IL-6, OSM, TGF-α, and VEGF-A. These findings suggest partial resolution of the acute inflammatory response with persistence of low-grade immune activation and tissue remodeling. However, the magnitude of these differences was modest, and their clinical relevance remains uncertain. In addition, sampling was performed at a single timepoint without assessment of symptoms, limiting interpretation of their relationship to persistent clinical sequelae. Further longitudinal studies incorporating clinical phenotyping will be required to determine the significance of these signals and their relevance to postviral recovery and potential long-term outcomes.

Severe viral infections are frequently characterized by dysregulated host responses, with excessive IL-6 and TNF production, pronounced interferon and chemokine signaling, and endothelial activation resembling a cytokine storm [31]. Dysregulated antiviral T-cell responses have been shown to play an important role in this process. Mpox infection and MVA-BN vaccination elicit robust OPXV-specific CD4^+^ and CD8^+^ responses [18]. The IFN-γ–chemokine signature that we observed in acute clade IIb mpox, alongside innate immune activation, is consistent with Th1 and cytotoxic T-cell engagement. Vaccine-primed T cells may constrain viral replication early in breakthrough infections, modifying systemic inflammation and potentially explaining the attenuated inflammatory profiles that we observed in vaccinated participants. Further studies incorporating cellular immune profiling will be important to better define the relationship among T-cell responses, inflammatory profiles, and clinical outcomes.

The absence of effective antiviral therapies for mpox highlights the need to explore host-directed approaches. The inflammatory pathways identified here represent potential targets for intervention; however, given the predominantly mild and self-limiting nature of clade IIb disease, the role of systemic immunomodulatory therapy is likely limited to select high-risk groups. Although agents such as BTK inhibitors have demonstrated in vitro activity against OPXVs and may modulate inflammatory pathways, clinical evidence is lacking [32]. These findings therefore provide a basis for considering targeted therapeutic approaches, particularly given the lack of effective antiviral strategies, but require validation in larger cohorts and in more severe disease.

This study has several limitations. The small sample size limited statistical power and adjustment for potential confounders; consequently, cluster-derived inflammatory profiles should be considered exploratory and may not generalize without validation in larger studies, and some findings from individual cytokine analyses with borderline adjusted *P* values may reflect variability given the sample size. The cohort comprised predominantly mild nonhospitalized clade IIb infection in adult males who were immunocompetent, limiting generalizability to severe disease, individuals with advanced HIV-associated immunosuppression, women, children, and populations in endemic settings, as well as to clade I infection. Variation in time from symptom onset across clusters may also contribute to differences in cytokine profiles, and findings may in part reflect temporal dynamics of the acute immune response. Sampling at single timepoints restricted analysis of temporal dynamics. Immune profiling was performed in plasma, which reflects systemic rather than local mucosal immune responses. Given that proctitis represents a mucosal inflammatory process, circulating cytokine signatures may therefore not fully capture immune activity at the rectal mucosa. Although concurrent sexually transmitted infections were excluded at the time of sampling, the contribution of unmeasured or recently resolved coinfections cannot be fully excluded. Finally, representation of PWH was limited to a single participant with a preserved CD4^+^ T-cell count, precluding conclusions regarding HIV-associated immunosuppression.

Despite these limitations, this study provides the first framework for understanding systemic immune profiles in clade IIb mpox. We identified a distinct inflammatory signature associated with mucosal disease in acute infection, alongside evidence of persistent low-grade immune activation and tissue remodeling during convalescence. These findings (1) underscore the central role of host inflammatory pathways in shaping clinical manifestations to acute viral infections, (2) suggest that vaccination may beneficially modulate host systemic inflammatory responses, and (3) highlight host inflammatory pathways as potential therapeutic targets. In the context of the ongoing outbreaks of clade I and II mpox globally, integrating cytokine and T-cell profiling into therapeutic and vaccine studies will be essential to refine risk stratification, guide future therapeutic strategies, and inform vaccine policy.

## Supplementary Material

ofag460_Supplementary_Data

## Supplementary Data

Supplementary materials are available at *Open Forum Infectious Diseases* online. Consisting of data provided by the authors to benefit the reader, the posted materials are not copyedited and are the sole responsibility of the authors, so questions or comments should be addressed to the corresponding author.
