# Supplementary material for: Distinct Inflammatory Immune Signature in Acute Mpox Associated With Proctitis
Source: Open Forum Infect Dis. 2026 Jul 23;13(8):ofag460. doi: 10.1093/ofid/ofag460 (PMC13448059; doi:10.1093/ofid/ofag460)
Supplement: Supplementary file 1 [file ofag460_supplementary_data.zip › Supplementary_materials_v2_clean.docx]

**Supplementary Materials**

**Table 1: Differential plasma cytokine levels in acute mpox compared with matched controls**

| **Cytokine** | **Acute Mpox**  **(pg/mL, n=16)** | **Matched Control (pg/mL, n=16)** | **Adjusted *p* value (FDR)** |
| --- | --- | --- | --- |
| **Innate immune activation** | | | |
| IL-6 | 5.24 (3.85–9.12) | 1.05 (0.85–1.57) | <0.001 |
| CXCL8 | 5.09 (4.13–10.16) | 4.23 (3.00–5.47) | 0.10 |
| IL-15 | 10.65 (7.51–14.83) | 14.14 (10.01–17.39) | 0.29 |
| IL-17A | 1.68 (0.84–2.86) | 0.55 (0.35–1.32) | 0.04 |
| IL-17C | 19.24 (16.00–28.53) | 22.81 (19.01–28.71) | 0.77 |
| IL-17F | 1.06 (0.73–1.62) | 1.53 (0.67–2.61) | 0.50 |
| IL-18 | 844.43 (743.56–1199.56) | 527.66 (372.58–716.69) | 0.01 |
| CCL2 | 469.12 (401.57–603.77) | 371.18 (294.57–491.21) | 0.04 |
| CCL3 | 10.26 (8.65–17.71) | 4.81 (3.83–6.10) | 0.009 |
| CCL4 | 105.9 (80.37–195.22) | 70.46 (50.91–116.06) | 0.10 |
| CCL7 | 1.24 (0.76–1.58) | 0.5 (0.36–0.66) | 0.01 |
| CCL8 | 67.35 (50.32–101.17) | 33.95 (24.25–39.97) | 0.005 |
| CXCL10 | 751.12 (440.20–976.56) | 153.54 (88.62–234.40) | 0.01 |
| CXCL9 | 530.16 (259.41–1060.45) | 121.58 (96.10–208.05) | 0.004 |
| CXCL11 | 251.54 (184.49–482.49) | 100.56 (73.70–126.76) | 0.01 |
| TNF | 35.69 (25.92–53.32) | 18.85 (16.50–22.05) | 0.003 |
| CSF1 | 186.19 (162.59–210.21) | 145.14 (130.97–159.60) | 0.01 |
| CSF2 | 0.28 (0.24–0.39) | 0.20 (0.16–0.24) | 0.03 |
| CSF3 | 130.38 (80.69–196.64) | 83.59 (70.42–118.94) | 0.08 |
| **TH1 activation** | | | |
| IL-1β | 0.59 (0.26–0.95) | 0.17 (0.11–0.22) | 0.01 |
| IL-2 | 0.06 (0.03–0.08) | 0.04 (0.01–0.05) | 0.07 |
| IFN-γ | 22.8 (2.79–99.99) | 0.35 (0.19–0.71) | <0.001 |
| **TH2 Activation** | | | |
| IL-4 | 0.04 (0.03–0.05) | 0.03 (0.01–0.04) | 0.08 |
| IL-13 | 0.54 (0.26–10.53) | 0.64 (0.17–2.09) | 0.47 |
| IL-33 | 0.27 (0.20–0.40) | 0.11 (0.07–0.17) | 0.01 |
| TSLP | 0.07 (0.05–0.11) | 0.06 (0.03–0.09) | 0.53 |
| CCL11 | 163.17 (107.3–183.69) | 152.84 (111.78–183.78) | 0.95 |
| CCL13 | 101.17 (80.00–118.67) | 64.53 (46.81–95.74) | 0.12 |
| **Immune regulation** | | | |
| IL-7 | 1.21 (0.81-1.65) | 0.93 (0.80-1.32) | 0.45 |
| IL-10 | 32.99 (21.03–49.37) | 12.23 (8.68–17.28) | 0.01 |
| IL-27 | 23.86 (16.1–52.95) | 13.53 (5.41–19.04) | 0.02 |
| LTA | 13.14 (12.05–18.62) | 10.94 (9.31–12.06) | 0.01 |
| CCL19 | 295.94 (236.01–408.78) | 117.98 (101.04–161.06) | 0.005 |
| **Endothelial activation and tissue repair** | | | |
| VEGF-A | 479.92 (347.37–631.46) | 258.96 (243.62–414.63) | 0.02 |
| CXCL12 | 230.07 (198.15–310.82) | 210.18 (184.13–236.64) | 0.19 |
| EGF | 31.17 (19.55–48.47) | 20.61 (15.36–26.04) | 0.09 |
| HGF | 543.04 (428.02–688.25) | 337.31 (264.53–439.36) | 0.01 |
| TGF-α | 10.44 (9.79–14.47) | 6.66 (6.00–8.46) | <0.001 |
| FLT3LG | 135.71 (110.09–161.89) | 116.63 (74.35–162.92) | 0.50 |
| OSM | 9.12 (7.55–18.29) | 2.78 (1.84–4.66) | 0.001 |
| **Matrix remodelling and inflammation** | | | |
| MMP1 | 1026.22 (733.13–1352.27) | 629.02 (526.53–1230.83) | 0.17 |
| MMP12 | 326.43 (246.02–401.31) | 183.72 (133.46–262.37) | 0.01 |
| **Metabolic oxidative stress and modulation** | | | |
| OLR1 | 154.46 (58.35–268.63) | 67.91 (41.18–101.21) | 0.02 |
| TNFSF10 | 650.91 (580.69–774.30) | 554.65 (478.29–685.90) | 0.21 |
| TNFSF12 | 660.22 (494.35–817.09) | 758.8 (576.59–867.98) | 0.47 |

Median (IQR) cytokine concentrations are shown for each group (pg/mL).

*p* values were derived using the Mann-Whitney U test, adjusting for multiple comparisons with Benjamini-Hochberg FDR correction.

**Table 2: Participant characteristics across inflammatory clusters in convalescent mpox and negative controls**

| **Cluster Demographics** | **Cluster 1 (*n*=12)** | **Cluster 2 (*n*=23)** | **Cluster 3 (*n*=6)** | ***p-*value** |
| --- | --- | --- | --- | --- |
| **Age (median (IQR))** | 32 (30-35) | 33 (30-41.5) | 37 (30-41.5) | 0.32* |
| **Male sex at birth (n(%))** | 12 (100%) | 23 (100%) | 6 (100%) | NA |
| **Race (n(%))**  **Caucasian** | 12 (100%) | 23 (100%) | 6 (100%) | NA |
| **HIV (n(%))**  **People with HIV** | 1 | 2 | 2 | 0.23** |
| **Group (n(%))**  **Convalescent Mpox**  **Negative Control** | 6  6 | 15  8 | 4  2 | 0.65** |
| **Mpox Parameters** | **Cluster 1**  **Convalescent Mpox (*n*=6)** | **Cluster 2**  **Convalescent Mpox (*n*=15)** | **Cluster 3**  **Convalescent Mpox (*n*=4)** | ***p*-value** |
| **Vaccination Status (n(%))**  **Vaccinated** | 1 | 2 | 0 | 0.71** |
| **Days from symptom onset (median (IQR))** | 396 (376-448) | 410 (277-441) | 290 (119-470) | 0.52* |
| **Overall Mpox Severity Scoring System (Mpox-SSS)^a^ (median (Min-Max))** | 7 (3-14) | 7 (3-14) | 8 (6-10) | 0.88* |
| **Median Score by category** |  |  |  |  |
| **Active lesion burden, number** | 1.5 | 1.5 | 2 |  |
| **Lesion burden, extent of body involvement** | 2 | 2 | 1.5 |  |
| **Confluent lesion or lesions with diameter >2 cm** | 0 | 0 | 0.5 |  |
| **Treatment for bacterial superinfection** | 0 | 0 | 0 |  |
| **Mucosal areas affected** | 1 | 1 | 1.5 |  |
| **Level of care** | 1 | 1 | 1 |  |
| **Pain, analgesia requirement** | 1 | 1 | 1 |  |

^a^Mpox Severity Scoring System (Mpox SSS: The sum of scores ranges from 1 through 23.(26)

**p*-value as derived from Kruskal-Wallis test

***p*-value as derived from Chi-Squared test

**Table 3: Plasma cytokine concentrations across acute, convalescent, and control groups**

| **Cytokine** | **Acute Mpox (pg/mL, n=16)** | **Convalescent Mpox (pg/mL, n=25)** | **Negative Controls (pg/mL, n=16)** | **Adjusted *p* value (FDR)** | **Acute vs Control (*p* value)** | **Acute vs Convalescent**  **(*p* value)** | **Convalescent vs Control**  **(*p* value)** |
| --- | --- | --- | --- | --- | --- | --- | --- |
| **Innate immune activation** | | | | | | | |
| IL-6 | 5.24 (3.85–9.12) | 1.95 (1.36–2.88) | 1.05 (0.85–1.57) | <0.001 | <0.001 | <0.001 | 0.039 |
| CXCL8 | 5.09 (4.13–10.16) | 3.61 (2.46–5.38) | 4.23 (3.00–5.47) | 0.075 |  |  |  |
| IL-15 | 10.65 (7.51–14.83) | 14.88 (11.46–19.12) | 14.14 (10.01–17.39) | 0.246 |  |  |  |
| IL-17A | 1.68 (0.84–2.86) | 1.36 (0.42–2.61) | 0.55 (0.35–1.32) | 0.094 |  |  |  |
| IL-17C | 19.24 (16.00–28.53) | 19.24 (15.24–36.71) | 22.81 (19.01–28.71) | 0.953 |  |  |  |
| IL-17F | 1.06 (0.73–1.62) | 1.51 (0.65–2.38) | 1.53 (0.67–2.61) | 0.685 |  |  |  |
| IL-18 | 844.43 (743.56–1199.56) | 495.87 (398.87–644.31) | 527.66 (372.58–716.69) | 0.004 | 0.005 | 0.001 | 0.725 |
| CCL2 | 469.12 (401.57–603.77) | 410.93 (335–517.66) | 371.18 (294.57–491.21) | 0.094 |  |  |  |
| CCL3 | 10.26 (8.65–17.71) | 4.50 (4.10–6.69) | 4.81 (3.83–6.10) | 0.001 | 0.001 | <0.001 | 0.873 |
| CCL4 | 105.9 (80.37–195.22) | 80.38 (59.94–105.63) | 70.46 (50.91–116.06) | 0.099 |  |  |  |
| CCL7 | 1.24 (0.76–1.58) | 0.40 (0.28–0.56) | 0.50 (0.36–0.66) | 0.004 | <0.001 | <0.001 | 0.381 |
| CCL8 | 67.35 (50.32–101.17) | 38.98 (26.14–47.58) | 33.95 (24.25–39.97) | 0.001 | <0.001 | 0.001 | 0.600 |
| CXCL10 | 751.12 (440.20–976.56) | 129.84 (81.20–189.76) | 153.54 (88.62–234.40) | 0.001 | 0.004 | <0.001 | 0.330 |
| CXCL9 | 530.16 (259.41–1060.45) | 103.26 (76.83–190.08) | 121.58 (96.10–208.05) | 0.001 | 0.001 | <0.001 | 0.511 |
| CXCL11 | 251.54 (184.49–482.49) | 69.29 (46.44–157.52) | 100.56 (73.70–126.76) | 0.004 | 0.012 | <0.001 | 0.413 |
| TNF | 35.69 (25.92–53.32) | 18.92 (17.21–26.37) | 18.85 (16.50–22.05) | 0.001 | <0.001 | <0.001 | 0.750 |
| CSF1 | 186.19 (162.59–210.21) | 156.63 (142.13–176.85) | 145.14 (130.97–159.60) | 0.012 | 0.002 | 0.027 | 0.195 |
| CSF2 | 0.28 (0.24–0.39) | 0.16 (0.12–0.23) | 0.20 (0.16–0.24) | 0.007 | 0.021 | 0.001 | 0.396 |
| CSF3 | 130.38 (80.69–196.64) | 80.4 (56.16–97.66) | 83.59 (70.42–118.94) | 0.084 |  |  |  |
| **TH1 activation** | | | | | | | |
| IL-1β | 0.59 (0.26–0.95) | 0.17 (0.15–0.29) | 0.17 (0.11–0.22) | 0.004 | 0.002 | 0.001 | 0.889 |
| IL-2 | 0.06 (0.03–0.08) | 0.03 (0.01–0.04) | 0.04 (0.01–0.05) | 0.025 | 0.040 | 0.003 | 0.511 |
| IFN-γ | 22.8 (2.79–99.99) | 0.26 (0.22–0.43) | 0.35 (0.19–0.71) | <0.001 | <0.001 | <0.001 | 0.597 |
| **TH2 Activation** | | | | | | | |
| IL-4 | 0.04 (0.03–0.05) | 0.03 (0.01–0.04) | 0.03 (0.01–0.04) | 0.071 |  |  |  |
| IL-13 | 0.54 (0.26–10.53) | 0.69 (0.24–1.82) | 0.64 (0.17–2.09) | 0.796 |  |  |  |
| IL-33 | 0.27 (0.20–0.40) | 0.11 (0.09–0.19) | 0.11 (0.07–0.17) | 0.010 | 0.003 | 0.004 | 0.660 |
| TSLP | 0.07 (0.05–0.11) | 0.04 (0.02–0.08) | 0.06 (0.03–0.09) | 0.569 |  |  |  |
| CCL11 | 163.17 (107.3–183.69) | 141.53 (124.77–201.86) | 152.84 (111.78–183.78) | 0.953 |  |  |  |
| CCL13 | 101.17 (80.00–118.67) | 76.87 (58.41–130.55) | 64.53 (46.81–95.74) | 0.227 |  |  |  |
| **Immune regulation** | | | | | | | |
| IL-7 | 1.21 (0.81-1.65) | 0.99 (0.72–1.82) | 0.93 (0.80-1.32) | 0.685 |  |  |  |
| IL-10 | 32.99 (21.03–49.37) | 14.13 (9.66–16.94) | 12.23 (8.68–17.28) | 0.007 | 0.002 | 0.004 | 0.549 |
| IL-27 | 23.86 (16.1–52.95) | 12.58 (10.60–22.67) | 13.53 (5.41–19.04) | 0.025 | 0.008 | 0.011 | 0.700 |
| LTA | 13.14 (12.05–18.62) | 10.28 (8.23–11.29) | 10.94 (9.31–12.06) | 0.001 | 0.005 | <0.001 | 0.442 |
| CCL19 | 295.94 (236.01–408.78) | 139.12 (105.28–176.22) | 117.98 (101.04–161.06) | 0.001 | <0.001 | <0.001 | 0.646 |
| **Endothelial activation and tissue repair** | | | | | | | |
| VEGF-A | 479.92 (347.37–631.46) | 367.97 (309.77–461.03) | 258.96 (243.62–414.63) | 0.025 | 0.004 | 0.311 | 0.032 |
| CXCL12 | 230.07 (198.15–310.82) | 241.51 (211.85–276.81) | 210.18 (184.13–236.64) | 0.390 |  |  |  |
| EGF | 31.17 (19.55–48.47) | 23.66 (16.98–37.00) | 20.61 (15.36–26.04) | 0.182 |  |  |  |
| HGF | 543.04 (428.02–688.25) | 374.80 (331.00–513.77) | 337.31 (264.53–439.36) | 0.007 | 0.001 | 0.011 | 0.235 |
| TGF-α | 10.44 (9.79–14.47) | 10.85 (7.84–13.49) | 6.66 (6.00–8.46) | 0.001 | <0.001 | 0.398 | 0.001 |
| FLT3LG | 135.71 (110.09–161.89) | 124.6 (104.79–158.47) | 116.63 (74.35–162.92) | 0.772 |  |  |  |
| OSM | 9.12 (7.55–18.29) | 5.31 (3.70–7.39) | 2.78 (1.84–4.66) | <0.001 | <0.001 | 0.006 | 0.029 |
| **Matrix remodelling and inflammation** | | | | | | | |
| MMP1 | 1026.22 (733.13–1352.27) | 767.03 (519.3–1202.34) | 629.02 (526.53–1230.83) | 0.276 |  |  |  |
| MMP12 | 326.43 (246.02–401.31) | 249.14 (210.27–333.70) | 183.72 (133.46–262.37) | 0.017 | 0.002 | 0.106 | 0.071 |
| **Metabolic oxidative stress and modulation** | | | | | | | |
| OLR1 | 154.46 (58.35–268.63) | 85.66 (68.18–148.46) | 67.91 (41.18–101.21) | 0.032 | 0.005 | 0.202 | 0.066 |
| TNFSF10 | 650.91 (580.69–774.30) | 548.13 (479.61–686.84) | 554.65 (478.29–685.90) | 0.138 |  |  |  |
| TNFSF12 | 660.22 (494.35–817.09) | 794.02 (697.50–1048.45) | 758.8 (576.59–867.98) | 0.110 |  |  |  |

Median (IQR) cytokine concentrations are shown for each group (pg/mL).

Global *p* values were obtained using the Kruskal-Wallis test with Benjamini-Hochberg FDR correction for multiple comparisons. Pairwise *p* values (acute vs control, acute vs convalescent, and convalescent vs control) are shown only for cytokines with significant global differences, based on Dunn's post-hoc tests.

**Figure 1. Scree plot of principal component analysis (PCA) in acute mpox and matched controls.**

**Figure 2**

The plot shows the proportion of variance explained by the first 10 principal components.

**Figure 2**

Boxplots display log10 transformed plasma levels of the 15 biomarkers that defined cluster 1, directly compared between all participants with acute mpox and matched controls. All 15 biomarkers were significantly elevated in acute mpox. *p* values were derived using the Mann-Whitney U test with Benjamini-Hochberg FDR correction for multiple comparisons.

**Figure 3. Scree plot of principal component analysis (PCA) in convalescent mpox and negative controls.**

The plot shows the proportion of variance explained by the first 12 principal components.

**Figure 4: Proposed model of mucosal-driven systemic inflammation in clade IIb mpox**

Schematic illustrating the hypothesised pathway linking mucosal mpox infection to systemic immune activation. Localised mucosal involvement, characterised by proctitis and epithelial barrier disruption, is proposed to initiate a distinct systemic inflammatory response marked by interferon-γ-skewed cytokine signalling, including IFN-γ, CXCL9/10/11 and IL-6, and the emergence of clustered inflammatory profiles. These systemic response may drive downstream tissue effects, including VEGF-A-mediated pathways, tissue repair and remodelling processes, and persistent low-grade inflammation observed during convalescence.
